# Increased beam attenuation and surface dose by different couch inserts of treatment tables used in megavoltage radiotherapy

**DOI:** 10.1120/jacmp.v12i4.3554

**Published:** 2011-11-15

**Authors:** Jan K.H. Seppälä, Jarmo A.J. Kulmala

**Affiliations:** ^1^ Department of Oncology and Radiotherapy Turku University Hospital Finland; ^2^ Cancer Center Kuopio University Hospital Finland

**Keywords:** beam attenuation, surface dose, couch insert, carbon fiber, skin sparing

## Abstract

The use of solid carbon fiber table materials in radiotherapy has become more common with the implementation of image‐guided radiotherapy (IGRT), since the solid materials give less imaging artifacts than the so‐called tennis racket couchtops. The downside of the solid carbon fiber couch inserts is that they increase the beam attenuation, resulting in increased surface doses and inaccuracies in determine the dose in the patient. The purpose of this study was to evaluate the interaction of 6 and 15 MV photons with eight different couch inserts. The presented results enable direct comparison of the attenuation properties of the studied couchtops. With a direct posterior beam the maximum attenuations reach 3.6% and 2.4% with 6 and 15 M V, respectively. The measured maximum attenuation by a couchtop with an oblique gantry angle was 10.8% and 7.4% at 6 and 15 MV energies, respectively. The skin‐sparing effect was decreased substantially with every couchtop. The highest increases in surface doses were recorded to be four‐ and threefold, as compared to the direct posterior open field surface doses of 6 and 15 MV, respectively. In conclusion, the carbon fiber tabletops decrease the skin‐sparing effect of megavoltage photon energies. The increased beam attenuation and skin doses should be taken into account in the process of treatment planning.

PACS number: 07.90.+c

## I. INTRODUCTION

In modern external beam radiotherapy (RT), the treatment planning is supposed to be dosimetrically and geometrically accurate, and individual patients should receive the prescribed dose within high accuracy. With the advent of new techniques in RT like image‐guided radiotherapy (IGRT), intensity‐modulated radiotherapy (IMRT) and volumetric‐modulated arc therapy (VMAT), the requirements for treatment tables have increased. To achieve the best possible treatment accuracy, the couch should be stiff in order to avoid any sagging of the treatment table. Also, table materials should not create any imaging artifacts that might decrease the setup accuracy of the treatment. However, with IMRT and VMAT, the treatment field directions are oblique, and more beams are required to penetrate the treatment couch so as to enhance the dose distribution in the patient. Therefore, the utilized couch inserts should have minimum attenuation to achieve precise dose accuracy and to maintain the skin‐sparing effect of megavoltage beams.

Light‐weight carbon fiber has been used in RT for making tabletops because carbon fiber has a high specific strength with high beam transmission when compared to other materials commonly used in RT devices.^(^ ^1^ ^)^ Despite the good characteristics of carbon fiber tabletops, the beam attenuation by the couch inserts can be significant. If the increased attenuation is not accounted for, this can result in underdosage of the target volume. Beam absorption by the tabletop can also be significant, thus increasing skin doses of patients which can be seen as an increase in skin toxicity.^(^ ^2^ ^)^ Photon beam attenuation properties of carbon fiber couch inserts have been studied by several investigators.^(^ ^3^ ^–^ ^13^ ^)^ Higgins et al.^(^ ^4^ ^)^ measured a relative increase of 375% in surface dose with 8 MV photons when a carbon fiber insert panel (Sinmed BV) was added to a $10\times 10\;{\text{cm}}^{2}$ beam. McCormack et al.^(^ ^6^ ^)^ measured a significant increase in beam attenuation ranging from 2.0% at 0° to 8.7% at 70° with the studied Sinmed BV Posisert carbon fiber couch insert, whereas Poppe et al.^(^ ^9^ ^)^ measured an attenuation of 2.7% at 0° with a RM2/4 tabletop in the $15\times 15\;{\text{cm}}^{2}$ beam. Mihaylov et al.^(^ ^10^ ^)^ measured the attenuation properties of an ExacTrac couchtop (in this study a BrainLAB couchtop is used) with 6 and 18 MV photon energies. With 6 MV they measured the beam attenuation to be 3.2% and 8.6% with beam incidence of 0° and 75°, respectively. The corresponding values of the study of Njeh et al.^(^ ^12^ ^)^ were 3.4% and 8.3% using the same couchtop.

The summary from the former studies is that the focus has mainly been on one couchtop at a time, and the studies have been performed with a range of energies, field sizes, gantry angles, and couchtops. Extensive studies with a variety of couch inserts have not been performed, which makes the direct comparison between different couch inserts difficult. In this study, we will evaluate the effects of the eight commercially available couch inserts on beam attenuation and skin dose with energies of 6 and 15 M V. Three of the studied couch inserts have not been investigated before. The dosimetric properties of various couchtops can be directly compared by using the results presented here. The results could be incorporated to a treatment planning system in order to model the dose in the patient more accurately, as described by Mihaylov et al. The presented data can also be used to avoid beam directions with the highest attenuation by a couchtop, so as to maintain the skin‐sparing effect of megavoltage radiation.^(^ ^6^ ^)^

## II. MATERIALS AND METHODS

**Figure 1:**
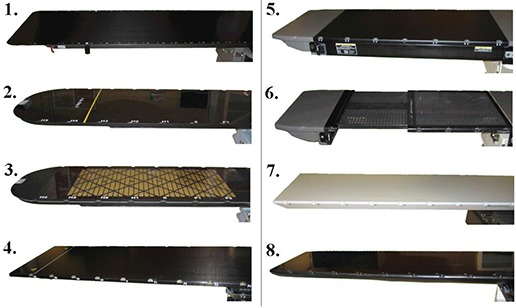
Couch inserts studied: 1) BrainLAB imaging couch top, 2) Qfix kVue Standard, 3) Qfix kVue DoseMax, 4) MEDTEC model MT‐IL 3303, 5) universal sandwich panel, 6) Varian grid insert, 7) DIGNITY AirPlate, 8) Varian Exact IGRT couchtop.

The effect of eight commercially available couchtops on photon beam attenuation and surface dose were measured with energies of 6 and 15 MV. The couchtops studied were: 1) BrainLAB imaging couchtop (BrainLAB, Heimstetten, Germany), 2) Qfix kVue Standard (WFR/Aquaplast, Avondale, PA), 3) Qfix kVue DoseMax (WFR/Aquaplast, Avondale, PA), 4) model MT‐IL 3303 (MEDTEC, Orange City, IA), 5) universal sandwich panel (solid insert) (Varian Medical Systems, Palo Alto, CA), 6) a standard Varian tennis racket grid insert (Varian Medical Systems, Palo Alto, CA), 7) DIGNITY AirPlate (ONCOlog Medical, Uppsala, Sweden), and 8) Varian Exact IGRT couchtop (Varian Medical Systems, Palo Alto, CA). Three of the studied couchtops are not so‐called imaging couchtops because of the metal rails on the side of the inserts. Those are the MEDTEC, universal sandwich panel, and the Varian grid insert couchtops. The images of the studied couch inserts are presented in Fig. 1. All the measured beams were from a Varian 2100 C/D linear accelerator with a dose rate of 400 MU/min.

### A. Beam attenuation measurements

**Figure 2:**
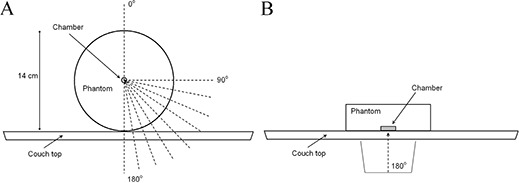
Measurement setup for couchtop beam attenuation (a) and surface dose (b) measurements. With attenuation measurements, the cylindrical ionization chamber was in the middle of the phantom. With surface dose measurements, the parallel‐plane chamber was facing the couchtop.

The effect of gantry angle on beam attenuation was studied with a field size of $10\times 10\;{\text{cm}}^{2}$. The measurements were performed with an ionization chamber (Farmer NE‐2571) at a depth of 7 cm in a cylindrical phantom made of PMMA. The measurement setup is illustrated in Fig. 2(a). The quantity of ionization produced in the ion chamber was measured with a Farmer 2570 Dosemeter (Nuclear Enterprises Ltd., Reading, UK). The phantom was aligned longitudinally on the treatment table and the isocenter was set to the center of the chamber. A reference value was measured with a direct anterior beam (gantry angle 0°). The beam attenuations by the eight couch inserts were measured every 5° at gantry angles between 90° and 180°. Couch rails, when present (kVue Standard, kVue DoseMax, universal sandwich panel, and the Varian grid insert), were removed from the beam path, since we wanted to study only the couch inserts. With the Varian grid insert, the measurement location was taken from the large grid area. With the Exact IGRT couchtop, the attenuation measurements were performed from the thinner part of the table, as the thickness of the couchtop is not constant in the longitudinal direction.

### B. Surface dose measurements

Relative surface doses were measured with a plane‐parallel ionization chamber (NACP‐02, IBA‐Scanditronix) in a water‐equivalent phantom with adequate backscattering material. The NACP‐02 chamber has a Mylar foil and graphite window with a combined thickness of 0.6 mm $\left(104\;\text{mg}/{\text{cm}}^{2}\right)$. The electrode spacing is 2.0 mm and the collecting electrode diameter is 10 mm. Measurements to quantify the increase in surface dose by a couch insert were performed with a direct posterior beam (gantry angle of 180°) with field sizes of $10\times 10\;{\text{cm}}^{2}$ and $20\times 20\;{\text{cm}}^{2}$. The phantom was aligned such that the chamber surface was facing the table, as illustrated in Fig. 2(b). The doses at maximum were measured for 6 and 15 MV and were set as reference doses. The surface dose was measured with no buildup material. When measuring the dose at maximum and the surface dose, the phantom was adjusted over the treatment couch so that the couch did not interfere with the measurements. All the measurements were performed at source to skin distance (SSD) 100 cm to the surface of the chamber. The increase in surface dose by a couch insert was calculated as the percentage of ionization compared to the ionization measured at the depth of dose maximum. The polarity correction factor was measured to be close to unity within the measurement uncertainty, and thus the measurements were performed with one polarity.

With the surface dose measurements, charged particle equilibrium does not exist because the ion chamber is located in the buildup region. This will cause perturbation effects in plane‐parallel ionization chambers mainly due to electron fluence perturbation through the chamber side wall.^(^ ^14^ ^)^ The perturbation effect was corrected by using a method proposed by Gerbi and Khan^(^ ^15^ ^)^ with constant restricted mass stopping power ratios between the point of measurement and dose maximum.

## III. RESULTS

### A. Beam attenuations

**Figure 3:**
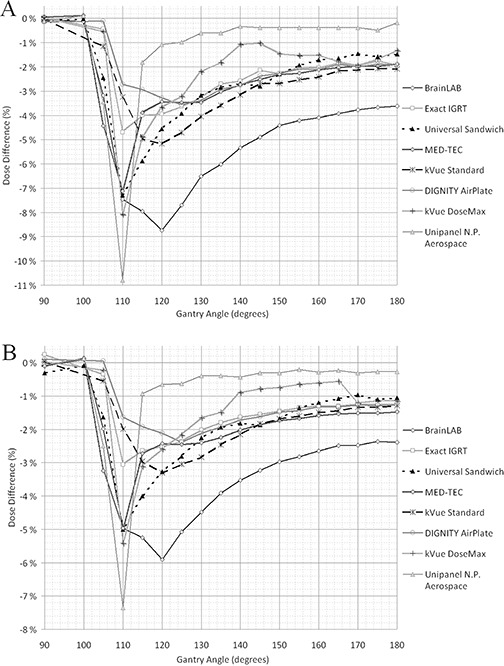
Beam attenuations of eight different couch inserts measured with energies of 6 (a) and 15 MV (b) with various gantry angles. The measured dose difference (%) is with respect to the dose measured with a gantry angle of 0°.

The beam attenuation of the studied couch insert as a function of gantry angle with energies of 6 and 15 MV are presented in Figs. 3(a) and (b), respectively. A summary of measured couch inserts and the corresponding maximum attenuation is given in Table 1. The highest maximum attenuation of 10.8% was recorded with the Varian grid insert with 6 MV and gantry angle of 110°. The beam attenuation with a direct posterior beam (180°) and the average attenuation between gantry angles of 100°–180° are also presented in Table 1. With a direct posterior beam, the highest attenuation was measured with BrainLAB couchtop. The corresponding beam attenuations were 3.6% and 2.4% with 6 and 15 MV, respectively. With energy of 6 MV the highest average attenuation (between gantry angles of 100°–180°) was recorded with BrainLAB (5.0%) and the lowest with the Varian grid insert (1.3%), respectively. The average attenuation was calculated since this might concern the treatments and the choice of a couch insert if the dose is delivered by VMAT. It should be noted that with couch inserts of kVue Standard, kVue DoseMax, universal sandwich panel, and Varian grid, the couch rails were moved away from the beam.

**Table 1:** The direct posterior (gantry angle 180°), maximum, and average beam attenuations (in percentage) measured with eight different couch inserts with energies of 6 and 15 MV. The average beam attenuation was calculated between gantry angles of 100°–180°. The couch inserts are in descending order with respect to attenuation with a direct posterior 6 MV beam.

	*6 MV*			*15 MV*		
	*GA 180°*	*Max.*	*Average (100°–180°)*	*GA 180°*	*Max.*	*Average (100°–180°*
BrainLAB	3.6	8.7	5.0	2.4	5.9	3.3
Qfix kVue Standard	2.1	5.2	2.9	1.4	3.3	1.9
MEDTEC	1.9	7.1	2.9	1.5	5.0	2.1
Varian Exact IGRT	1.9	4.7	2.4	1.3	3.1	1.6
DIGNITY AirPlate	1.9	3.6	2.3	1.2	2.4	1.5
Universal Sandwich Panel	1.5	7.3	2.8	1.0	5.0	2.0
Qfix kVue DoseMax	1.3	8.1	2.2	1.0	5.4	1.4
Varian Grid Insert	0.3	10.8	1.3	0.2	7.4	0.9

### B. Increase in surface dose

The measured surface doses relative to the dose maximum with different couch inserts in the beam are given in Table 2. The measured open field surface doses at the effective depth of $1.04\;\text{mg}/{\text{cm}}^{2}$ with $10\times 10\;{\text{cm}}^{2}$ field size were 44.3% and 27.6% with 6 and 15 MV photons, respectively. When the perturbation effect was corrected, the corresponding percentage of ionizations at the measured water‐equivalent depth of 1.04 mm decreased to 35.2% and 21.0%. With couchtops that were not uniform in shape (Exact IGRT) or that had a grid structure (kVue DoseMax and the Varian grid insert), the presented results are the average measured surface doses in three different locations on the couch. The largest increase in surface dose was observed with the BrainLAB couchtop with a dose of 98.6% of the dose maximum with $10\times 10\;{\text{cm}}^{2}$ and 6 MV. The corresponding lowest surface dose was 61.2% measured with the Varian grid insert. When the increase in surface dose was compared to the corrected open field surface doses, the most significant escalation was observed with 15 MV beam of $10\times 10\;{\text{cm}}^{2}$ with the BrainLAB couchtop giving an increase of 400%. With 6 MV, the corresponding increase in surface dose was 280%.

**Table 2:** Measured surface doses relative to the dose maximum $\left(D_{\max}\right)$ (in percentage) with different couch inserts in the beam with energies of 6 and 15 MV. Measurements were performed with $10\times 10\;{\text{cm}}^{2}$ and $20\times 20\;{\text{cm}}^{2}$ open fields with a direct posterior beam (gantry angle 180°). The corrected surface dose takes into account the perturbation effect of the used plane‐parallel ionization chamber. The couch inserts are in descending order with respect to relative surface dose.

	*6 MV*		*15 MV*	
	$10\times 10\;cm^{2}$	$20\times 20\;cm^{2}$	$10\times 10\;cm^{2}$	$20\times 20\;cm^{2}$
Measured Surface Dose	44.3	53.4	27.6	39.1
Corrected Surface Dose	35.2	44.3	21.0	32.6
Dose at Dmax	100.0	100.0	100.0	100.0
BrainLAB	98.6	99.4	84.5	89.9
Varian Exact IGRT	90.8	94.0	69.6	77.7
Universal Sandwich Panel	90.2	93.2	69.2	76.9
MEDTEC	90.1	93.4	69.1	77.3
Qfix kVue Standard	88.5	92.1	66.9	75.2
DIGNITY AirPlate	86.0	89.8	66.0	74.3
Qfix kVue DoseMax	75.1	80.9	52.8	63.0
Varian Grid Insert	61.2	69.0	41.0	51.7

## IV. DISCUSSION

The use of megavoltage beams in RT has decreased the skin erythema, fibrosis, and desquamation when compared to orthovoltage beams.^(^ ^16^ ^)^ The skin‐sparing effects of megavoltage beams means that the skin no longer has to be considered as a limiting organ for curative RT treatments. However, after the implementation of solid carbon fiber couchtops, skin reactions have increased in our clinical practice when treating through the couch material.^(^ ^2^ ^)^ This is of concern because not only do some carbon fiber couchtops increase the skin dose of the patients,^(^ ^17^ ^)^ but they also attenuate the penetrating radiation beam. An understanding by clinicians of the increased beam attenuation and surface doses by the couch inserts is important to avoid underdosage of the target volume and skin toxicity. The photon beam attenuation and increased surface doses caused by different couch inserts have to be modeled to ensure precise dose accuracy and to quantify the skin doses of patients.

The photon beam attenuation properties of different couchtops have been studied by various authors. Njeh et al.^(^ ^12^ ^)^ measured the attenuation by the BrainLAB imaging couchtop. They recorded the highest attenuation of 8.3% with a gantry angle of 120° and an attenuation of 3.4% with a direct posterior beam with 6 MV photons and a field size of $10\times 10\;{\text{cm}}^{2}$. These results are comparable to our measurements with corresponding attenuations of 8.7% and 3.6%, respectively. The 6 MV photon measurements of Vanetti et al.^(^ ^18^ ^)^ of the Varian Exact IGRT couchtop (the thinner part) showed attenuations of 2.3% and 3.1% with gantry angles of 180° and 135°, respectively. Our corresponding measured attenuations were 1.9% and 2.7%. Butson et al.^(^ ^8^ ^)^ used GAFCHROMIC EBT films to measure the increase in skin dose with a Varian carbon fiber grid couchtop. With a direct posterior 6 MV beam and a field size of $10\times 10\;{\text{cm}}^{2}$, the skin dose increased from 27% with no buildup to 55%. Our results from the measurements (presumably with the same couch model) at the effective depth of 1.04 mm were 35.2% and 61.2%, respectively.

The measured average beam attenuation between gantry angles of 100°–180° was lowest with the Varian grid insert (1.3%). This couchtop, however, has a grid‐like structure, resulting in imaging artifacts (if using kV‐image guidance). This couchtop, as well as three other couchtops in this study, has rails to support the couch insert. These rails were moved away from the beam when the measurements were performed. The average beam attenuation with a solid carbon fiber couchtop without the supporting rails was lowest with DIGNITY AirPlate (2.3%) and highest with BrainLAB couchtop (5.0%). From the measurements we can conclude that, with grid couchtops, the surface dose from a direct posterior beam is the lowest. However, the side bars of these couches are more solid and thus absorb the dose less uniformly, which is seen as the highest attenuation of 10.8% with a gantry angle of 110°. The grid pattern also worsens the image quality taken on the treatment machines and the grid couch inserts also need the supportive rails. The solid carbon fiber couchtops are more rigid than the grid‐based couchtops and the attenuation is more homogeneous as a function of gantry angle. Unfortunately, the beam attenuation is also higher and thus the surface doses are also increased. It should be noted that some of the couchtops have different couch extensions (e.g., for bracing the head^(^ ^12^ ^)^). They also have different beam attenuation properties than the couchtop itself and were not included in this study.

Representative depth of acute and late skin radiation reactions for erythema and subcutaneous fibrosis are considered to be between 0.1 and 2 mm.^(^ ^19^ ^,^ ^20^ ^)^ The depth of radiation‐induced skin reactions is consequently close to the water‐equivalent window thickness of 1.04 mm of the used plane‐parallel ionization chamber. The measurements on the surface area with a parallel‐plane ionization chamber, however, give an overestimation of the measured surface dose because of the perturbation effect of the chamber. When the correction factor was applied,^(^ ^15^ ^)^ the measured $10\times 10\;{\text{cm}}^{2}$ open field surface doses decreased from 44.3% and 27.6% to values of 35.2% and 21.0% with 6 and 15 MV photons, respectively. The corrected values are similar to the surface doses of 33.3% and 24.4% measured by Ishmael Parsai et al.^(^ ^21^ ^)^, with an extrapolation chamber at a depth of 0.5 mm with the same field size and with energies of 6 and 10 MV, respectively. When reviewing the increased surface doses by the couch inserts in Table 2, it should be noted that the ionization is measured with a direct posterior beam. The magnitude of skin dose will increase with the angle of incidence of the beam.^(^ ^8^ ^)^ That is, the presented values are the best‐case scenarios with respect to skin doses when treating through the couchtop. The reader should also keep in mind that, in this study, we only investigated the couch inserts and not any immobilization devices or possible couch rails which also contribute to the beam attenuation.

In some treatment planning systems, the treatment couch can be modeled to account for increased beam attenuation. This enhances the dose accuracy in the target volume and in the critical organs.^(^ ^10^ ^,^ ^13^ ^,^ ^18^ ^)^ Unfortunately, this does not diminish the problem of the loss of skin‐sparing effect, but the knowledge of the increased skin dose could be used to alter the treatment plan. Also, if the treatment couch attenuation can be accounted for, the patient positioning has to be with respect to the couch center; otherwise erroneous dose distributions could be generated if the beam was only partially attenuated by the couch insert.

The limitation of this study is that the output of the linear accelerator was proposed to be constant with different gantry angles. However, this inaccuracy should be under 1% and the possible error would appear as the same with every couchtop.^(^ ^12^ ^)^ The reader should also bear in mind that the photon beam attenuations measured with different gantry angles in this study are only from one point of a measurement (7 cm above the couch surface), and that the attenuation depends also on the height of the measurement relative to the couch insert. In addition, the attenuation and the increase in the surface dose by the couchtops were measured only with few field sizes and with the same SSD, as the attenuation and the effect on the surface dose are also dependent on those aspects.^(^ ^12^ ^)^ The estimated uncertainty of the NACP chamber measurements in the buildup region is mainly a sum of inaccuracies in the Gerbi and Khan correction method^(^ ^15^ ^)^ and the difference in the mass stopping‐power ratios with different depths. When surface dose measurements are corrected with the Gerbi and Khan method and are compared with Monte Carlo simulations, the difference is found to be less than 4%.^(^ ^22^ ^)^ The correction factor also changes as a function of depth, and is reported to be insignificant at depths deeper than 1 mm.^(^ ^22^ ^)^ The dependency of the stopping‐power as a function of depth for 6 and 15 MV photon beam is calculated to be less than 1%.^(^ ^22^ ^)^ When the inaccuracies in the determination of the ionization current (less than 1%) are also considered, we can estimate that the maximum deviation in the surface dose measurements is less than 5%.

## V. CONCLUSIONS

In this study, the dosimetric properties of eight commercially‐available treatment tables were investigated. In conclusion, the carbon fiber couch inserts decrease the skin‐sparing effect of megavoltage beams. The increased beam attenuation and skin doses of patients should be taken into account in the process of treatment planning. As a result of this study, the attenuation properties of the studied couch inserts can be compared directly. The data presented here can be used in treatment planning systems to account for increased surface doses and photon beam attenuation.

## ACKNOWLEDGMENTS

The authors thank Tony Shepherd for his comments on the manuscript, and the Radiotherapy Department of Turku University Hospital for the use of its facilities.
